# Quality Assessment and Ripeness Prediction of Table Grapes Using Visible–Near-Infrared Spectroscopy

**DOI:** 10.3390/foods12122364

**Published:** 2023-06-14

**Authors:** Fengjiao Ping, Jihong Yang, Xuejian Zhou, Yuan Su, Yanlun Ju, Yulin Fang, Xuebing Bai, Wenzheng Liu

**Affiliations:** 1 College of Enology, Northwest A&F University, Yangling 712100, China; 2 Shaanxi Engineering Research Center for Viti-Viniculture, Yangling 712100, China

**Keywords:** Vis-NIR spectroscopy, table grape, soluble solids, total acid, ripeness

## Abstract

Ripeness significantly affects the commercial values and sales of fruits. In order to monitor the change of grapes’ quality parameters during ripening, a rapid and nondestructive method of visible-near-infrared spectral (Vis-NIR) technology was utilized in this study. Firstly, the physicochemical properties of grapes at four different ripening stages were explored. Data evidenced increasing color in redness/greenness (a*) and Chroma (C*) and soluble solids (SSC) content and decreasing values in color of lightness (L*), yellowness/blueness (b*) and Hue angle (h*), hardness, and total acid (TA) content as ripening advanced. Based on these results, spectral prediction models for SSC and TA in grapes were established. Effective wavelengths were selected by the competitive adaptive weighting algorithm (CARS), and six common preprocessing methods were applied to pretreat the spectra data. Partial least squares regression (PLSR) was applied to establish models on the basis of effective wavelengths and full spectra. The predictive PLSR models built with full spectra data and 1st derivative preprocessing provided the best values of performance parameters for both SSC and TA. For SSC, the model showed the coefficients of determination for calibration ($R_{Cal}^{2}$) and prediction ($R_{Pre}^{2}$) set of 0.97 and 0.93, respectively, the root mean square error for calibration set (RMSEC) and prediction set (RMSEP) of 0.62 and 1.27, respectively; and the RPD equal to 4.09. As for TA, the optimum values of $R_{Cal}^{2}$, $R_{Pre}^{2}$, RMSEC, RMSEP and RPD were 0.97, 0.94, 0.88, 1.96 and 4.55, respectively. The results indicated that Vis-NIR spectroscopy is an effective tool for the rapid and non-destructive detection of SSC and TA in grapes.

## 1. Introduction

Grape (*Vitis vinifera* L.) is a commonly cultivated fruit crop around the world, which is rich in sugar, organic acids, vitamins, minerals, and other nutrients [1]. In addition to its nutritional value, grapes also possess significant medicinal properties [2]. Ripeness is a key quality parameter that determines the commercial value and sales of grapes [3] and significantly affects the timing of grape harvesting [4]. During the entire ripening process, grapes undergo biochemical and physiological changes, i.e., softening, pigment accumulation, and flavor and aroma formation [5]. For these changes, ripeness is often evaluated indirectly based on parameters such as soluble solids content (SSC) and total acid (TA) levels, as they reflect grape quality and ripeness, with low SSC and high TA values indicating unripe and less flavorful grapes [6,7,8]. However, the traditional method of testing these parameters is predicated on destroying the integrity of the whole grape bunch and requires a large number of samples, which is time consuming and uneconomical, as well as requires professional operators with expertise [9].

At present, spectroscopy technology, especially for near-infrared (NIR) or visible–near-infrared (Vis-NIR), is extensively utilized in the area of agricultural product detection because of its rapid, nondestructive, low cost, and reliability characteristics [10,11]. A number of studies have described the use of spectral detection methods to analyze fruit maturity. For instance, Cirilli et al. [12] evaluated the ripening evolution of olive fruit in terms of physical and chemical change features by using a portable NIR-AOTF device (1100–2300 nm). Musingarabwi et al. [13] utilized Fourier-transform NIR spectroscopy to analyze grape berries at different development stages, and achieved satisfactory results. Pu et al. [14] utilized Vis-NIR hyperspectral imaging techniques to measure the contents of SSC and PH, and discriminated the maturity of lychee fruits based on the results. Escribano et al. [15] successfully predicted SSC and dry matter content in sweet cherries using NIR spectroscopy and demonstrated its potential for grading fruit based on eating quality with commercial cultivars. Additionally, Xiao et al. [16] used Vis-NIR (400–1100 nm) and NIR (900–2500 nm) spectroscopy based on the full-band and selected wavelengths to discriminate five ripening stages of different grape cultivars. The SSC-based model using CARS-SV-DA obtained satisfactory discrimination accuracy for ‘Manicure Finger’ and ‘Ugni Blanc’ (90% and 100%), respectively. Pourdarbani et al. [17] used Vis-NIR (400–1000 nm) spectroscopy to investigate Fuji apple maturity stages and found that the correlation coefficients of determination of starch content, acidity, and tissue firmness were 0.940, 0.919, and 0.800, respectively. Pissard et al. [18] assessed a portable NIR spectrometer for detecting the quality of apple fruit, and found that the coefficient of determination and the root mean square error of cross-validation values of SSC were 0.91 and 0.57, respectively. Vega-Castellote et al. [19] successfully predicted SSC of watermelons during their development on the vine using portable NIR spectrometers, which was a significant effort towards the in-situ nondestructive test for the fruits with characteristics of thick and large size. Fatchurrahman et al. [20] studied the comparison performance of Vis-NIR (400–1000 nm) and NIR (900–1700 nm) hyperspectral imaging for prediction of nutritional quality of goji berry in four maturity stages. They found that NIR obtained better results for the determination of vitamin C (*R*^2^_pred_ = 0.91), and Vis-NIR performed better in phenols, SSC, and TA prediction. As can be seen, all these studies indicated that Vis-NIR or NIR spectroscopy shows excellent potential in determining the ripeness of various fruits. However, according to our literature search, there are few reports on table grapes and the prediction of SSC, TA, and maturity index using Vis-NIR spectroscopy. Furthermore, it is challenging to achieve a handheld portable operation in determining fruit maturity among the majority of previous studies, which required laboratory instruments.

In this study, we utilized a miniature spectrometer with fiber optic signal couplers to perform Vis-NIR spectroscopy for handheld operation. Fiber optics offers high transmission rates and flexibility for transient acquisition of spectra and piggybacking of spectral acquisition systems in large fields [21]. To our knowledge, the literature currently lacks systematic research applications that aimed at determining the maturity of table grapes through analysis of SSC and TA contents. In this study, we applied Vis-NIR spectroscopy to capturing the change in quality indices of table grapes at various maturity stages for modeling analysis. Concurrently, the method of partial least squares regression (PLSR) was adopted to assess the maturity of table grapes.

The objectives of this study were to (1) analyze the changes in color, texture, SSC, and TA content of grapes at different maturity stages; (2) develop prediction models using Vis-NIR spectra for SSC and TA in grapes; and (3) evaluate the application efforts of the models in measuring grape ripeness.

## 2. Materials and Methods

### 2.1. Sample Collection

In this study, table grapes (*Vitis labruscana* L. cv. Kyoho) were collected from the Cao Xinzhuang vineyard (34°18′0″ N; 108°5′23.9″ E, Yangling, China) in four development stages (i.e., color, hardness, elasticity) during July and August of 2021 by experienced growers. The grapes without visible damage or disease were selected. The sampling process followed a predetermined principle, wherein two rows of grapes were randomly selected as fixed sampling points, and five berries were randomly picked from the top, middle, and bottom of each cluster using the five-point sampling method [16]. The sampling process lasted for about two months and grape samples were collected between 8:00 and 10:30 am on each sampling day. Four batches of experimental samples were collected in different stages of maturity, with approximately 800 berries in each batch.

**Figure 1:**
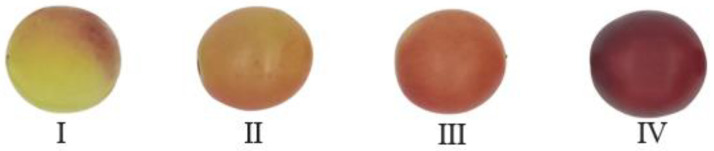
Appearance of grapes at different stages of ripeness.

As shown in Figure 1, grapes in stage I were less mature fruits with greenish skin color and harder texture; grapes in stage II were slightly mature grapes with larger red skin areas and harder texture; grapes in stage III presented full red color and softer texture; grapes in stage IV were fully mature fruits with the skin color turning to purple and the softest texture. From each batch of 800 berry samples, grapes were further subdivided into 40 groups with similar physiological status to improve the accuracy of the model [22]. Fifteen berries from each group were used to measure the physicochemical properties, and the other five berries were used to obtain spectral data.

### 2.2. Vis-NIR Reflectance Spectral Data Acquisition

**Figure 2:**
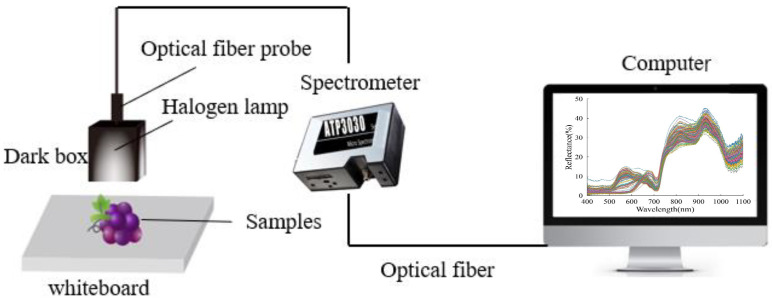
Reflectance spectral acquisition system.

The Vis-NIR reflectance spectral data of grape samples were collected using an ultra-high-resolution fiber optic spectrometric system (OPTOSKY Technology Co., Ltd., Xiamen, China). The wavelength region scanned was from 200 to 1100 nm with a sampling interval of 0.5 nm. A halogen lamp with 12 V Bulb/HL2000 was utilized as light source, and was installed at the internal part of the dark box, which also consisted of the handheld light unit (Figure 2). Each sample was placed on a whiteboard and placed in the dark box to obtain spectral data. Note that the distribution of SSC/TA in the whole grape berry is inhomogeneous. In order to minimize the influence of the inhomogeneous distribution, the spectra were measured at three different positions on each sample around the grape berry’s equator (~120°) and perpendicular to the longitudinal axis. These three spectra for each berry sample were then averaged into one spectrum, which was used as the final spectral data of the tested sample for the establishment of calibration models.

### 2.3. Measurement of Physicochemical Parameters

#### 2.3.1. Color Testing

The skin color of grape samples was tested using a colorimeter (Puyun Electronics Co., Ltd., Shenzhen, China). Measurements were performed in the CIELab system. L* (lightness), a* (redness/greenness), and b* (yellowness/blueness) were determined around the equatorial region [23]. C* (Chroma) and h* (Hue angle) were calculated according to Equations (1) and (2) [23]. Five repeated measurement values for each sample group were calculated and analyzed.

(1) $$C^{*2}=\sqrt{a^{*2}+b^{*2}}$$

(2) $$h^{*2}=\tan^{-1}\left(\frac{b^{*}}{a^{*}}\right)$$

#### 2.3.2. Texture Measurement

The texture characteristics of grape samples were measured using a texture analyzer (Stable Micro Systems, London, UK). Grape samples were compressed twice in succession by 30% of their equatorial height at a rate of 0.5 mm/s [24]. In this paper, the hardness, elasticity, and chewiness of the table grapes were determined. Each texture indicator test was repeated five times and the mean value was obtained.

#### 2.3.3. SSC and TA Determination

The real quality parameters of grape sample, soluble solids (SSC), and total acid (TA) were obtained from traditional destructive tests. This step was conducted immediately after collecting the spectral data of the samples. The grape juice, excluding skin and seeds, was used to measure SSC using a digital brix refractometer (Guangzhou Ai Measure Intelligent Technology Co., Ltd., Guangzhou, China). TA content was determined using a Mettler automatic potentiometric titrator (Mettler-Toledo International Inc., Zurich, Switzerland). The average of the SSC/TA values that were obtained from three repeated tests was calculated and recorded as the final reference value.

### 2.4. Data Analysis and Model Establishment

#### 2.4.1. Spectral Data Preprocessing

In spectral analysis, it is an important step to preprocess the obtained spectral data with appropriate preprocessing methods to enhance the accuracy of prediction models [6]. There are six common preprocessing methods, including standard normal variate (SNV), multiple scattering correction (MSC), 1st derivative, 2nd derivative, S-G smoothing, and S-G smoothing + 1st derivative, which were applied to deal with the spectral data and helped us choose the best one.

#### 2.4.2. Effective Wavelength Selection

The spectral data generally contain large amounts of redundant information and noise, which affect the robustness of models and increase the computation time [10,25]. To simplify the volume of models and highlight the useful spectral information, a suitable method is necessary to obtain effective wavelengths. The competitive adaptive weighting algorithm (CARS) can eliminate non-informative variables, filter out the wavelengths with the largest correlation coefficients, and enhance the predictive performance of models [26]. Therefore, CARS was used to pick out the most independent variables and establish models in a fast and accurate way.

#### 2.4.3. Model Establishment Method

Partial least squares regression (PLSR) is a commonly used method for statistical analysis, which has strong processing ability on multi-collinearity of linear regression and effectively reduces the negative influence of information loss and obtains a better modeling effect [27,28]. Thus, PLSR was applied to establish the prediction models required for Vis-NIR spectroscopy-based analyses in this study.

#### 2.4.4. Model Performance Evaluation

Model performance was assessed using the statistical terms of coefficients of determination for the calibration set ($R_{Cal}^{2}$) and the prediction set ($R_{Pre}^{2}$), the root mean square error for the calibration set (RMSEC) and the prediction set (RMSEP), and the residual prediction deviation (RPD). These assessment parameters were calculated as follows:

(3) $$R_{Cal}^{2},\;R_{Pre}^{2}=1-\frac{\sum_{i=1}^{n}{\left(y_{i}-{\hat{y}}_{i}\right)}^{2}}{\sum_{i=1}^{n}{\left(y_{i}-\bar{y}\right)}^{2}}$$

(4) $$\mathrm{RMSEC},\;\mathrm{RMSEP}=\sqrt{\frac{\sum_{i=1}^{n}{\left[y_{i}-{\hat{y}}_{i}\right]}^{2}}{n}}$$

(5) $$\mathrm{RPD}=\frac{\sqrt{\frac{1}{n}\sum_{i=1}^{n}{\left(y_{i}-\frac{1}{n}\sum_{i=1}^{n}y_{i}\right)}^{2}}}{\sqrt{\frac{1}{n}\sum_{i=1}^{n}{\left({\hat{y}}_{i}-y_{i}\right)}^{2}}}$$

where *n* is the number of samples used in the calibration set; *y_i_* is the measured value of samples’ quality parameters; $\bar{y}$ is the average of the measured values of samples’ quality parameters; and *ŷ_i_* is the predicted value of samples’ quality parameters. In general, the spectral model’s prediction outcome is optimal when both $R_{Cal}^{2}$ and $R_{Pre}^{2}$ reach their highest values, while RMSEC and RMSEP are minimized. Additionally, a desirable condition is that the value of RPD exceeds three [29].

In this paper, the whole process of data analysis and model establishment was conducted using MATLAB 2016a (The Mathworks, Natick, MA, USA) and SPSS 26 (IBM, Armonk, NY, USA) on a laptop (Matebook X Pro, Huawei Technologies Co., Ltd., Shenzhen, China). The analysis of variance (ANOVA) was conducted to compare the data along ripening stages. Differences at *p* < 0.05 were considered statistically significant by Duncan’s new multiple range test.

## 3. Results and Discussion

### 3.1. Analysis of Physical and Chemical Indicators of Grapes

#### 3.1.1. Color Analysis

Fruit skin color is often considered a prominent and highly utilized quality indicator that significantly affects consumer’s acceptance [30]. A previous study found that the varying contents of natural chlorophyll and pigments lead to the fruit color change during ripening [31]. Table 1 shows the color transformation traits of grapes during the maturation process. The values of L*, a*, and b* were within the ranges of 25.15–35.80, 0.77–8.02, and 2.07–7.31, respectively. The L* and b* values decreased significantly (*p* < 0.05) from ripening stage I to IV, while the a* values increased significantly (*p* < 0.05) from ripening stage II to IV. The aforementioned findings indicated that as the grape’s growth time increased, there was a gradual reduction in the green portion of the peel and an increase in the red portion aligning with the observations depicted in Figure 1. This could be attributed to the degradation of chlorophyll and the accumulation of anthocyanin [32]. Nonetheless, a notable increase in the a* values was not observed between stage I and II, indicating a gradual color change in grapes during this period. This phenomenon may be attributed to water stress resulting from dry weather conditions [33]. At the same time, the slow increase in red tones during the pre-color transition period is probably due to the low accumulation of monomeric anthocyanins during the ripening stage from I to II. Overall, the color transformation during the later stages of fruit ripening is more prominent compared to the initial stages [34].

Chroma (C*) is used to determine the different degree of a hue in comparison to the grey color, which is considered as the quantitative attribute of colorfulness. A previous study found a significant correlation between C* and the color intensity of fruits [35]. The C* value increased significantly (*p* < 0.05) with the development of ripeness. Therefore, it can be seen that the higher ripeness degree resulted in a more intense purple-red color on the grape skin. The hue angle (h*) is generally represented the color in degrees, i.e., 0° for red, 90° for yellow, 180° for green, and 270° for blue [35]. According to the data, there was an obvious reduction (from 83.24 to 14.24) in h* values in grapes between the ripening stages from I to IV. It indicated that the grape color changed from yellow-green to purplish-red with the development of ripeness, and the ripening stage significantly influenced the color of grapes.

#### 3.1.2. Texture Characteristics

Texture properties of fruits are the key factors of ripeness of fruits and are influenced by changes in cell wall structure and composition during the ripening process [36]. The texture changes of grape samples were monitored in the ripening stages I, II, III, and IV, as shown in Table 2. With the progress of grape ripening, the hardness decreased sharply (*p* < 0.05) from stage I to III, while there was a slow hardness decrease in the ripening stages III to IV. The average hardness of grapes in stage I was 1435.87 g, then it gradually fell to 967.49 g when grapes proceeded to stage IV. The gradual softening of the fruit during ripening was evidenced by the study of Liu et al. [37]. Previous studies indicated that the softening of fruit was due to enzyme mediated alteration in the structure and composition of cell wall, partial or complete solubilization and depolymerization of cell wall polysaccharide like pectin and celluloses [38,39].

Furthermore, the elasticity of the grapes in the ripening stages of I to II presented significant difference (*p* < 0.05) from the stages III to IV, and the chewiness in stage I differed significantly (*p* < 0.05) from the stages II to IV. Additionally, the chewiness decreased sharply once stage I was completed, which was consistent with the trend in hardness, proving a positive correlation (*p* < 0.01) between hardness and chewiness [40]. The above changes were probably caused by the softening of grapes during the ripening period. Consequently, there was a close relationship between the texture and the maturity stages of grapes.

#### 3.1.3. Soluble Solids and Total Acid Analysis

The soluble solids (SSC), titratable acid (TA), and the ratio of SSC to TA (SSC/TA) are the critical quality indicators that affect the taste index and the consumer’s acceptance of fruits [23,41]. About 90% of the soluble solids in grapes are glucose, sucrose, and fructose, which together contribute to the overall sweetness of the fruit.

**Figure 3:**
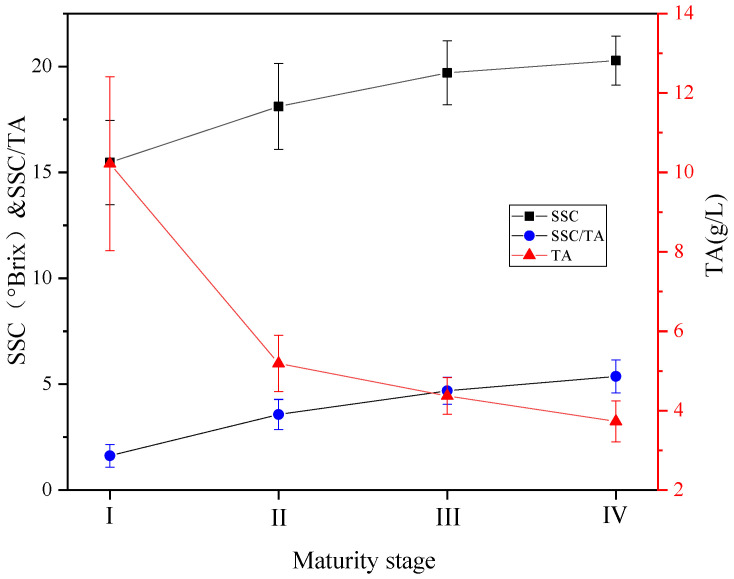
Variation of SSC, TA and SSC/TA in grapes during ripening. Note: Vertical bars denote SD.

Figure 3 shows the values of SSC, TA, and SSC/TA in the four ripening stages of table grapes. The SSC and TA contents ranged from 15.47 to 20.28% and 3.73 to 10.22 g/L, respectively, consistent with previous research conducted by Lima et al. [42]. As expected, SSC and SSC/TA increased while TA decreased during grapes’ ripening, indicating a gradual accumulation of sugars and degradation of organic acids. Moreover, there were significant changes (*p* < 0.05) from stage I to II. It may be due to the sudden increase in temperature during the ripening stages from I to II, which resulted in the high-speed degradation of organic acids. However, there was no longer a significant variation in SSC from stage III to IV, indicating that the grapes had reached the peak level of maturity. As can be seen, the higher the maturity, the sweeter the grapes taste. Therefore, harvesting grapes in the optimal stage of ripeness becomes crucial to ensure the production of flavorful fruits [23]. In summary, physiological changes in SSC and TA content are closely correlated with grape ripening.

### 3.2. Spectral Model Establishment

#### 3.2.1. Spectral Profile

**Figure 4:**
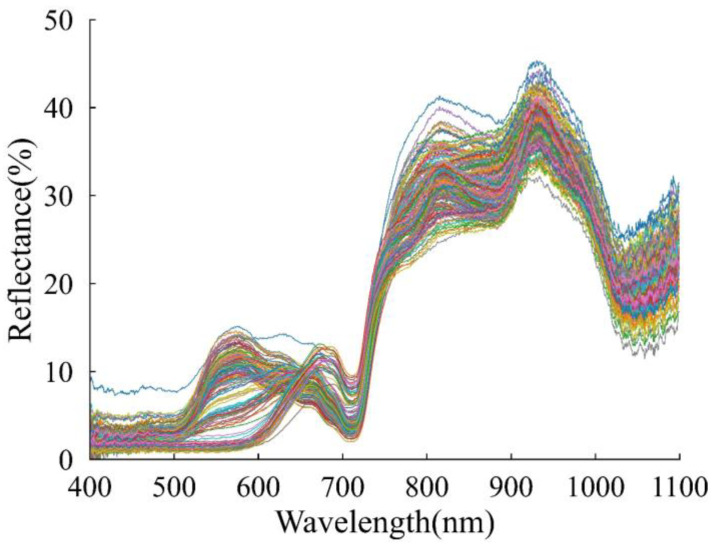
Original NIR spectral curves of grape samples.

Figure 4 presents the diffuse reflectance spectra of 159 groups of grapes in varying levels of ripeness. Despite instances of overlap and crossovers in these spectra, consistent trends were observed overall. Notable differences were observed within the spectral range of 510–720 nm, which can be attributed to variations in skin color due to different stages of grape ripening [43]. There was an evident absorption peak around 740 nm, which could be associated with the absorption of carotenoids [44] and chlorophylls [45]. The spectra were characterized by absorption bands at 900–970 nm, probably due to the absorption of phenols, anthocyanins, cellulose, and sucrose. Similar research results have been reported by Escribano et al. [15] and Hernández-Sánchez et al. [46]. In addition, absorption bands were observed in a spectral range of 1050–1100 nm, which is probably related to water and vitamin ingredients [20,47].

#### 3.2.2. Effective Wavelength Selection

**Figure 5:**
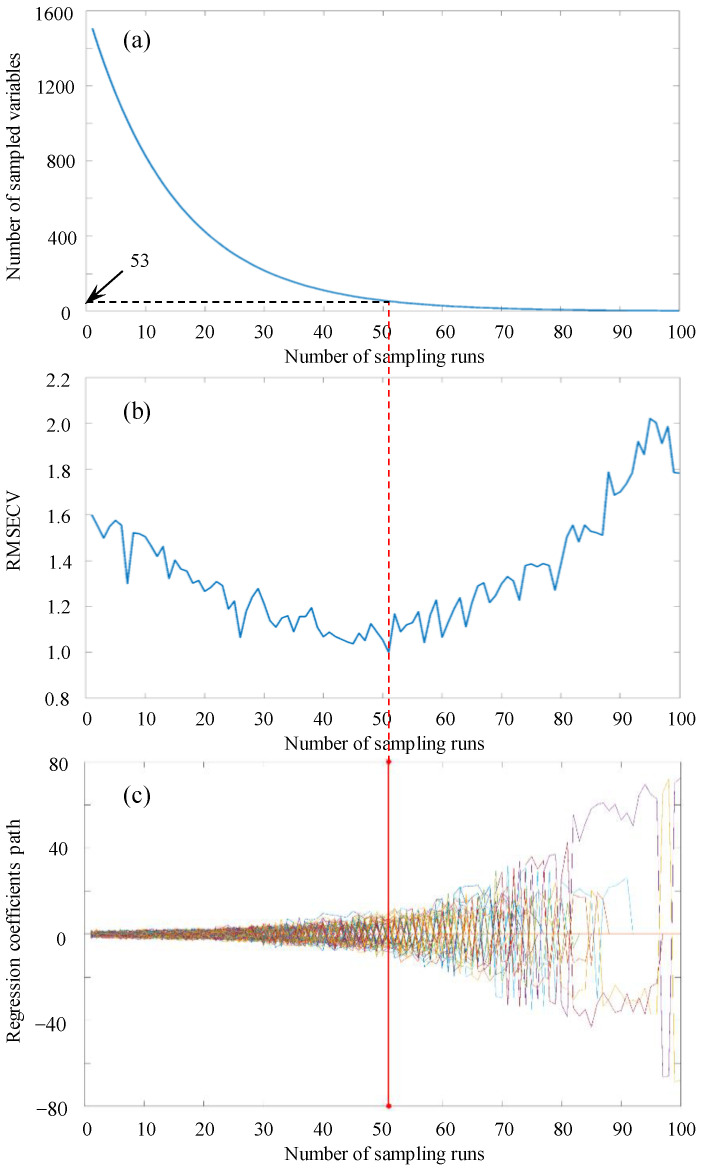
Effective selection of spectral data based on CARS, (**a**) the number of sampled variables, (**b**) 10-fold RMSECV values, and (**c**) the regression coefficient path of each variable with the increase in the number of sampling runs. The line (marked as red line) denotes the optimal point where the lowest RMSEV value is obtained.

The raw spectral data contained 1507 individual wavelengths. In order to eliminate the useless information within the full spectral data, CARS was used to select the effective wavelengths. Figure 5 illustrates the CARS variable selection process of grape samples with full spectral data. The number of Monte Carlo sampling runs was set to 100, and the significance of variables was determined by 10-fold cross validation [48].

As can be seen from Figure 5a, there appeared a sharp fall at the beginning stage and then turned to relative stable in the number of sampled bands with increasing sampling runs. It showed the refined and fast selection of the CARS algorithm. Figure 5b illustrates the changing trend of 10-fold RMSECV values as the number of sampling runs increased. The RMSECV values represented a descending tendency in a gentle way as the number of sampling runs increased from 1 to 51, which could be attributed to the gradual elimination of uninformative variables. Then, the RMSECV values began to increase as valuable spectral variables were removed. The minimal RMSECV value was obtained in the 51st sampling run where a red line is shown (Figure 5c), and was used to select a group of effective variables [26]. In Figure 5c, the path of the regression coefficient for each variable at different sampling runs is presented with each line. Variables with larger regression coefficients were deemed more suitable for selection. Therefore, effective spectral variables can be acquired for each sampling run by analyzing the regression coefficients [43]. Based on the CARS method, 53 effective wavelengths were selected from the full spectra (Table 3), which resulted in a reduction of more than 94% in data volume.

#### 3.2.3. Prediction Model Establishment

PLSR was utilized to explore the complicated spectral data and establish prediction models for evaluating SSC and TA. Additionally, PLSR models were established using both full spectra and effective wavelengths, and six common preprocessing methods were utilized. Results of PLS models based on full spectra and effective wavelengths are shown in Table 4 and Table 5, respectively.

As can be seen in Table 4, different spectral preprocessing methods had a different impact on the PLSR model results based on the full spectra. In relation to the prediction of SSC, apart from the preprocessing methods of 2nd derivative (RPD < 3) and SG smoothing ($R_{Cal}^{2}$ < 0.9), the other preprocessing algorithms yielded higher accuracy in model prediction. This result might be due to the involved noise information during the 2nd derivative and SG smoothing preprocessing progress [29]. When combining SG smoothing with 1st derivative, we observed an improvement in modeling performance compared to using SG smoothing alone. Additionally, the results of spectral modeling using SNV and MSC showed similar outcomes, albeit both performed less effectively due to $R_{Cal}^{2}$ < $R_{Pre}^{2}$ [49]. When comparing the different spectral preprocessing modeling results, the performance of prediction model was found to be optimal when 1st derivative was adopted for spectral data preprocessing. 

For TA prediction, except for 1st and 2nd derivative, other preprocessing methods did not significantly improve the model prediction accuracy ($R_{Cal}^{2}$ < $R_{Pre}^{2}$). Compared to the 2nd derivative, the modeling results using the 1st derivative as a preprocessing step showed better outcomes with higher values of $R_{Cal}^{2}$, $R_{Pre}^{2}$ and RPD, as well as lower values of RMSEC and RMSEP. In conclusion, 1st derivative was the most suitable preprocessing method in PLSR prediction models of SSC and TA based on full spectra.

As represented in Table 5, the performance of PLSR models, based on effective wavelengths, varied depending on the spectral preprocessing methods used. In the case of SSC prediction, the most accurate PLSR model was achieved by combining SG smoothing with 1st derivative with $R_{Cal}^{2}$ and $R_{Pre}^{2}$ values of 0.95 and 0.92, respectively, and RMSEC RMSEP of 0.81 and 1.01, respectively. Additionally, RPD was 5.47. In the case of TA, the best PLSR prediction model was achieved when MSC was used with $R_{Cal}^{2}$ = 0.94, $R_{Pre}^{2}$ = 0.94, RMSEC = 1.59; RMSEP= 1.78, and RPD = 4.16.

**Figure 6:**
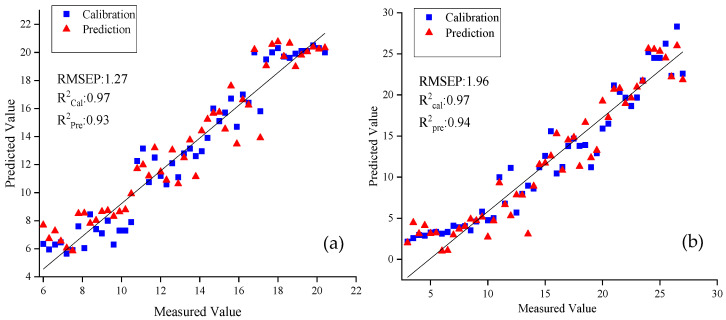
Scatter plots of measured versus predicted values of SSC (**a**) and TA (**b**) for the calibration (■) and the prediction (▲) sets.

After conducting a thorough analysis of the PLS models' results, considering the findings from Table 4 and Table 5, a comprehensive conclusion was drawn: although the prediction models development using full spectra costed more computation time, models based on full spectra performed better than those based on effective wavelengths, with the majority of $R_{Pre}^{2}$ and RPD values being over 0.92 and 4, respectively. Consequently, the combination of 1st derivative preprocessing and full spectra was applied to establish the best PLS spectral prediction models of SSC and TA in this study. Figure 6 shows the scatter plots of predicted versus measured SSC and TA values of grape samples. The regression line, represented by a solid line, visually depicts the correlation between the measured and predicted values. Notably, Figure 6a demonstrates a good fitting performance for SSC. Conversely, Figure 6b reveals slightly poorer prediction results for TA at lower values. This discrepancy may be attributed to the physiological maturity of the grape samples and the stabilization of TA contents [7]. Nevertheless, despite this observation, the sample in general exhibit a relatively uniform distribution close to the regression line. These findings suggest that PLS models can accurately forecast SSC and TA contents of grapes throughout ripening period.

## 4. Conclusions

In this study, the changes in physicochemical properties of grapes during ripening were explored, providing a scientific foundation for the maturity grading of grapes during this period. Results showed that as ripening advanced, the values of color in a* and C*, SSC content and SSC/TA ratio significantly (*p* < 0.05) increased, while color in L*, b* and h*, hardness, chewiness, flexibility and TA content gradually decreased. Subsequently, the spectral prediction models of SSC and TA in grapes were established. The results indicated that the model prediction performance is significantly affected by the spectral preprocessing methods. The combination of 1st derivative preprocessing and full spectra yielded the most effective PLS spectral prediction models of SSC and TA, providing a reference and theoretical basis for rapid and nondestructive detection. However, further research is necessary to develop more accurate and robust prediction models. This can be achieved by increasing the sample size and incorporating more grape cultivars. Additionally, the utilization of additional statistical methods, such as linear regression analysis (e.g., multiple linear regression, principle component analysis) and deep learning techniques (e.g., support vector machine, artificial neural network), can help improve the prediction accuracy and model establishment. Furthermore, it is crucial to recalibrate the prediction models when applying them to grapes harvested in different years, which will enhance the reliability and feasibility of the developed models.

## Figures and Tables

**Table 1:** Quantitative analysis of the color in table grapes during ripening.

Ripening Stage	L*	a*	b*	C*	h*
I	35.80 ± 3.87 _a_	0.77 ± 2.08 _c_	7.31 ± 1.51 _a_	5.77 ± 1.59 _c_	83.24 ± 14.36 _a_
II	32.31 ± 2.85 _b_	1.90 ± 2.55 _c_	4.80 ± 1.66 _b_	6.26 ± 0.98 _c_	66.30 ± 25.84 _b_
III	27.69 ± 2.88 _c_	5.82 ± 2.54 _b_	3.26 ± 1.68 _c_	7.24 ± 1.32 _b_	32.84 ± 23.34 _c_
IV	25.15 ± 1.46 _d_	8.02 ± 1.36 _a_	2.07 ± 0.81 _d_	8.32 ± 1.46 _a_	14.24 ± 4.14 _d_

Note: Data are expressed as mean ± SD (standard deviation). Different letters at the same column denote that the values are significant different (*p* < 0.05).

**Table 2:** Differences in Hardness, Flexibility, and Chewiness of grapes during ripening.

Ripening Stage	Hardness	Chewiness	Flexibility
I	1435.87 ± 285.19 _a_	238.64 ± 100.00 _a_	0.60 ± 0.05 _b_
II	1075.31 ± 110.70 _b_	196.79 ± 38.98 _b_	0.62 ± 0.03 _a_
III	982.84 ± 134.65 _bc_	192.09 ± 55.96 _b_	0.60 ± 0.06 _ab_
IV	967.49 ± 99.82 _c_	165.17 ± 22.56 _b_	0.57 ± 0.02 _c_

Note: Data are expressed as mean ± SD (standard deviation). Different letters at the same column mean the values are significant differenct (*p* < 0.05).

**Table 3:** Effective wavelengths selection using CARS.

Method	Effective Wavelengths (nm)								
CARS	38	61	62	73	306	307	403	404	405
	425	426	427	702	755	756	757	758	759
	775	787	788	834	835	836	908	909	910
	958	1087	1099	1100	1108	1123	1127	1131	1150
	1189	1262	1275	1297	1298	1312	1326	1344	1358
	1390	1391	1403	1417	1431	1453	1468	1499	

**Table 4:** Comparison of PLS models based on full spectra with different preprocessing methods.

Parameter	Pre-Processing Methods	Calibration		Prediction		RPD
		$R_{Cal}^{2}$	RMSEC	$R_{Pre}^{2}$	RMSEP	
SSC	SNV	0.91	1.13	0.96	0.88	5.44
	MSC	0.91	1.13	0.96	0.88	5.33
	1st derivative	0.97	0.62	0.93	1.27	4.09
	2nd derivative	0.94	0.80	0.82	1.94	2.70
	S-G Smoothing	0.89	1.31	0.94	1.06	4.43
	**S-G Smoothing +** **1st derivative**	**0.95**	**0.78**	**0.92**	**1.33**	**3.95**
TA	SNV	0.95	1.25	0.96	1.57	5.43
	**MSC**	**0.95**	**1.44**	**0.96**	**1.45**	**5.22**
	1st derivative	0.97	0.88	0.94	1.96	4.55
	2nd derivative	0.95	1.16	0.93	2.18	4.10
	S-G Smoothing	0.92	1.80	0.96	1.24	5.43
	S-G Smoothing + 1st derivative	0.95	1.25	0.96	1.56	5.43

Note: $R_{Cal}^{2}$ and $R_{Pre}^{2}$ are the coefficients of determination for calibration set and prediction set, respectively; RMSEC is the root mean square error of calibration; RMSEP is the root mean square error of prediction; RPD is the residual prediction deviation. Bold values indicate the best developed model.

**Table 5:** Comparison of PLS models based on effective wavelengths with different preprocessing methods.

Parameter	Pre-Processing Methods	Calibration		Prediction		RPD
		$R_{Cal}^{2}$	RMSEC	$R_{Pre}^{2}$	RMSEP	
SSC	SNV	0.93	1.18	0.88	1.26	4.13
	MSC	0.92	0.84	0.90	1.10	5.66
	1st derivative	0.88	1.26	0.89	1.45	3.52
	2nd derivative	0.84	1.40	0.79	2.12	2.45
	S-G Smoothing	0.87	1.43	0.91	1.28	3.68
	**S-G Smoothing +** **1st derivative**	**0.95**	**0.81**	**0.92**	**1.01**	**5.47**
TA	SNV	0.91	1.93	0.88	2.32	3.11
	**MSC**	**0.94**	**1.59**	**0.94**	**1.78**	**4.16**
	1st derivative	0.81	2.46	0.78	3.40	2.38
	2nd derivative	0.88	2.15	0.84	2.92	2.86
	S-G Smoothing	0.90	1.79	0.92	2.18	3.15
	S-G Smoothing + 1st derivative	0.93	1.81	0.89	2.12	4.70

Note: $R_{Cal}^{2}$ and $R_{Pre}^{2}$ are the coefficients of determination for calibration set and prediction set, respectively; RMSEC is the root mean square error of calibration; RMSEP is the root mean square error of prediction; RPD is the residual prediction deviation. Bold values indicate the best developed model.

## Data Availability

The related data and methods are presented in this paper. Additional inquiries should be addressed to the corresponding author.
